# Molecular Profiling of Inflammatory Bowel Disease: Is It Ready for Use in Clinical Decision-Making?

**DOI:** 10.3390/cells8060535

**Published:** 2019-06-04

**Authors:** Ho-Su Lee, Isabelle Cleynen

**Affiliations:** 1 Laboratory of Complex Genetics, Department of Human Genetics, KU Leuven, Herestraat 49 – box 610, 3000 Leuven, Belgium; hosu.lee@student.kuleuven.be; 2 Department of Biochemistry and Molecular Biology, University of Ulsan College of Medicine, Seoul 05505, Korea

**Keywords:** inflammatory bowel disease, genetics, transcriptomics, molecular profiling

## Abstract

Inflammatory bowel disease (IBD) is a heterogeneous disorder in terms of age at onset, clinical phenotypes, severity, disease course, and response to therapy. This underlines the need for predictive and precision medicine that can optimize diagnosis and disease management, provide more cost-effective strategies, and minimize the risk of adverse events. Ideally, we can leverage molecular profiling to predict the risk to develop IBD and disease progression. Despite substantial successes of genome-wide association studies in the identification of genetic variants affecting IBD susceptibility, molecular profiling of disease onset and progression as well as of treatment responses has lagged behind. Still, thanks to technological advances and good study designs, predicting phenotypes using genomics and transcriptomics in IBD has been rapidly evolving. In this review, we summarize the current status of prediction of disease risk, clinical course, and response to therapy based on clinical case presentations. We also discuss the potential and limitations of the currently used approaches.

## 1. Introduction

The inflammatory bowel diseases (IBD), which include Crohn’s disease (CD) and ulcerative colitis (UC), are chronic remittent inflammatory disorders of the gastrointestinal tract, with a multifactorial etiology. They are clinically heterogeneous and encompass a wide range of subtypes, each with its own pattern of disease behavior, location, and outcome. In general, clinical characterization of patients with IBD has been adopted by the Montreal classification [1]. While this classification system is reliable to classify phenotypes of IBD, its association with disease severity and ability to predict the clinical course is limited [2,3]. Accurately predicting the most likely clinical course is necessary to separate patients on the basis of their disease prognosis. Patients with a poor prognosis will indeed benefit most from an early aggressive therapy and require more frequent follow-up. In addition to, and in part because of, these diverse clinical phenotypes and severity levels, there is substantial heterogeneity in a patient’s individual response to therapy and in potential adverse events. A better prediction of therapeutic response and/or side effects will thus enable the selection of more optimal treatments and reduce the risk of adverse events.

For these reasons, genetic and molecular profiling of IBD has been at the forefront of IBD research for many years. Thanks to scientific and technological advancements in data generation, much progress has been made. For example, genome-wide association studies (GWAS) have identified ca. 240 genetic loci associated with IBD [4,5], have helped us to understand the genetic basis of IBD, and have indicated some key pathways [6]. Similar studies have been undertaken to also understand how these variants are associated with sub-phenotypes of the disease, treatment response, or side effects to therapy. However, these studies have not provided consistent and robust results, which are required for translation to the clinic. Transcriptomic studies have addressed the same questions, and thanks to modern technologies and good study designs, predicting phenotypes using genomics and transcriptomics in IBD has been rapidly evolving.

In this review, we focus on the current progress in genetic and transcriptomic profiling of IBD. We start from hypothetical clinical case presentations, each with a question related to disease risk, disease progression, response to therapy, or adverse events related to therapy, and provide an answer based on the present state of knowledge. We also discuss the potential and limitations of IBD molecular profiling for these purposes.

## 2. The Predictability of IBD Risk

### 2.1. Case 1: 34-Year-Old Female with a 10-Year History of Ileal CD

*Case description:* A 34-year-old female with CD remained in remission for three years. She wished to become pregnant and was concerned about the risk for her child to also develop CD. She carried a homozygous mutation within *NOD2* (p.L1007fsX).

The incidence (rate of newly diagnosed cases) and prevalence (number of patients at a specific time point) of IBD vary worldwide. In Europe, the annual incidence is 0–13 per 100,000 inhabitants for CD and 1–24 per 100,000 for UC. The prevalence for CD is 1–322 per 100,000 inhabitants, and that for UC is 5–505 per 100,000 inhabitants. Similar figures are reported for North America [7]. Low-incidence areas include Asia and South America, with a crude annual overall incidence value per 100,000 individuals of 1.37 for IBD in Asia [8]. This incidence is increasing, even in populations that were previously considered low-risk groups. This is partly due to the modernization and industrialization of these countries [9]. This means a baseline lifetime risk of 1.3% for someone of European ethnicity, like our case 1 described above [10].

Case 1 has an affected first-degree relative, which is the strongest established risk factor for IBD (Table 1). Studies of familial risk in IBD have reported a 4–15 times greater risk for IBD in first-degree relatives [11,12,13,14]. If both parents have IBD, the lifetime risk for their offspring is even thought to be over 30% [15,16]. The rate of family history in CD and UC has been reported to be approximately between 2% and 15% and is usually higher in patients with CD than in patients with UC [12,14,17]. Also, an additive risk increment for CD in subjects from multiple-affected families was reported per additional affected relative [18]. A shared genetic background as well as environmental factors could lead to the familial aggregation often seen in IBD.

Large-scale international genetic studies have identified more than 240 susceptibility loci harboring common variants (minor allele frequency >1–5%) associated with IBD [4,5]. These loci typically only have low to intermediate penetrance, which reflects the complexity and polygenic nature of IBD. The strongest risk is seen for *NOD2* variants, with an odds ratio estimated between 2.1 and 3.0 in Europeans [4]. Most other susceptibility variants show odds ratios in the order of 1.1–1.5 [6]. Even when carrying the ‘high-risk’ *NOD2* variant, with an average life-time risk for IBD of 1.3%, this represents an increase of lifetime risk to 3.9% (life-time risk 1.3% multiplied with the effect size of 3.0) (Table 1). This is still fairly low and also means that 96.1% of individuals carrying this risk variant will never develop the disease. It is also important to highlight the effect of demographic, environmental, lifestyle, and clinical risk factors. It is often underappreciated that many other risk factors have effect sizes that are like those of risk alleles discovered by GWAS, such as smoking, which is known to increase risk of CD (effect size of 1.8 [19]).

**Figure 1:**
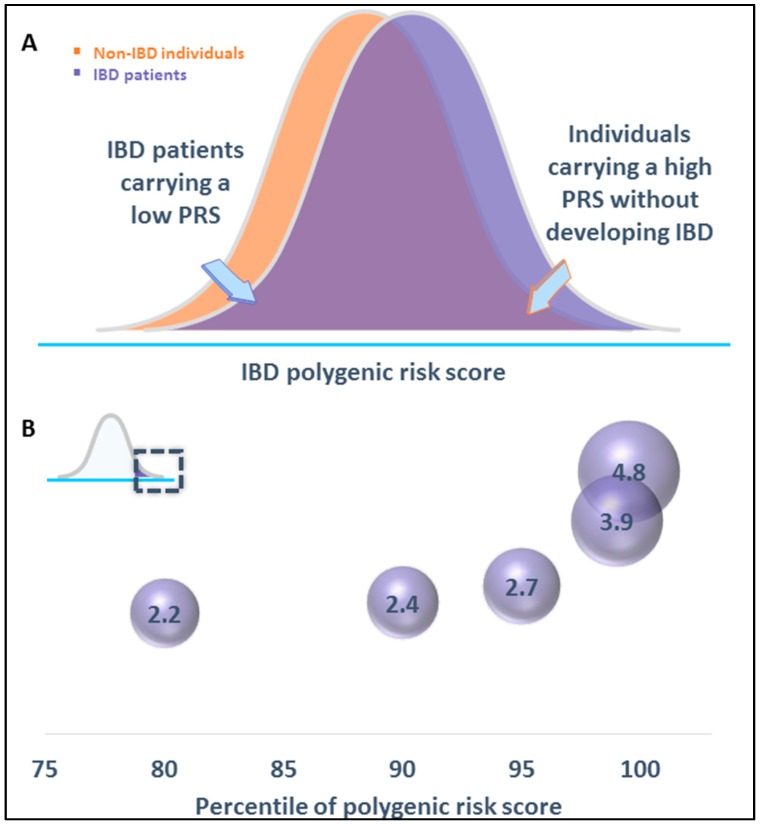
The prediction of IBD risk based on the polygenic risk score (PRS). (**A**) Histogram of IBD PRS distribution in controls and patients with IBD. Patients tend to have larger risk scores than controls, as seen by the shift to the right of the patient distribution (purple) compared to the control distribution (salmon), although the distributions of patients and controls tend to overlap for the most part. Also, it is equally possible to see patients with IBD but with a low IBD PRS (blue arrow on the left), as there are controls with high IBD PRS that do not develop IBD (orange arrow on the right). Thus, calculating someone’s PRS is only able to tell something about that persons’ risk to get the disease (compared to the general population), but not whether he/she will get the disease. (**B**) Risk gradient for IBD in the high polygenic risk score area (20% highest scores, dashed box). The ascertainment of individuals with high polygenic risk score in a population may provide an opportunity to identify the individuals with the highest genetic risk. On this figure, the increased fold-risk is indicated for individuals in the top 20%, 10%, 5%, 1%, and 0.5% of the distribution based on estimates from Khera et al. [20]. The size of the circles and the numbers inside indicate the odds ratios. The top 1% of PRS thus has 3.9-fold risk (compared with the remainder 99% of the population), which represents an increase of IBD risk to 5.1% (with a lifetime IBD risk of 1.3%). The utility of polygenic risk score-based risk estimations is thus currently limited by the relatively small effect sizes.

Individual risk variants are thus not helping us in predicting the risk to develop IBD. Considering the polygenic nature of IBD, a combined genetic burden instead of individual risk variants could maybe be used to identify individuals at clinically significant increased risk of IBD. This overall genetic burden is calculated as polygenic risk scores (PRS), summing risk alleles across all susceptibility loci, each weighted by the strength of their association. The use of PRS has become increasingly popular in the context of complex diseases [20] and has been applied also to IBD [20,21,22]. Patients with IBD tend to have larger risk scores on average, but the distributions of scores in patients and the general population overlap for the most part (Figure 1) [20,21,22]. In familial IBD, a higher burden of common risk variants has been observed: unaffected first-degree relatives of IBD patients have a higher PRS than the general healthy population, although their PRS is lower than that of individuals diagnosed with IBD [23,24]. Therefore, there is a clear genetic basis in the observed increase of IBD in families; however, the established susceptibility single-nucleotide polymorphisms (SNPs) seem to account for only a portion of the observed heritability of IBD. Thus, the utility of PRS for diagnosing the disease is currently limited even in familial IBD. It should also be noted that PRS is only able to tell something about the risk of one getting the disease (compared to the general population) but not about whether one will get the disease (Figure 1). At present, genetic profiling might help to identify individuals at high risk, though there currently are no established effective prevention strategies available. The latter are in general required to define genetic testing as useful and ethical. However, PRS-based risk estimates are beginning to show promise in their ability to identify possibly misdiagnosed patients. Using a genetic risk score with CD-versus-UC association weights and looking at patients at either extreme of the distribution could identify patients more likely to require a revised diagnosis (CD instead of UC or UC instead of CD) at follow-up [25].

### 2.2. Case 2: Two-Year-Old Child Presents with Very Early Onset (VEO) IBD

*Case description:* A toddler presented with severe, bloody diarrhea at two years of life. His endoscopy demonstrated severe pancolitis with duodenal involvement. He developed a perianal fistula two months later. He remained unresponsive to medical and surgical therapy.

In addition to common genetic variants conferring polygenic susceptibility to IBD as described above, there is a small fraction of patients with monogenic IBD. These patients can present with a spectrum of rare genetic disorders, including immune deficiencies [26]. Around 50 single genes causing these cases of VEO-IBD or IBD-like intestinal inflammation have been identified, such as *IL10R* and *XIAP* [26,27,28].

Many VEO-IBD patients are refractory to conventional treatment, as is also seen in our case patient. A correct and early diagnosis is crucial, as these patients may be treated by hematopoietic stem cell transplantation or targeted therapy (e.g., IL1-receptor antagonist anakinra for IL10R-deficient patients [29]). To identify the cause of the disorder, genetic analysis is required. This analysis can include whole-exome sequencing or targeted sequencing (e.g., Mendeliome sequencing [30,31,32]) of the patient—and his parents—to identify the causal mutation. In addition, when a diagnosis based on genetic analysis can be made, these families can get genetic counselling and thus receive advice on the risk of recurrence and on medical, psychological, and familial implications. Hence, the application of next-generation sequencing has become a widespread diagnostic tool in children who present with VEO-IBD during infancy or early childhood [28].

## 3. The Predictability of IBD Disease Progression

### 3.1. Case 3: 24-Year-Old Female with a One-Year History of Ileal CD

*Case description:* This non-smoking patient was diagnosed with CD with nonstricturing/nonpenetrating behavior, after which azathioprine (2.5 mg/kg/day) was initiated and maintained. Oral prednisone was commenced nine months before due to a flare-up and was tapered off three months later. However, two weeks before the visit, her abdominal pain and diarrhea recurred. She was worried she would have to start more aggressive treatments or would eventually need surgery. Can we predict her clinical course on the basis of molecular profiling?

**Figure 2:**
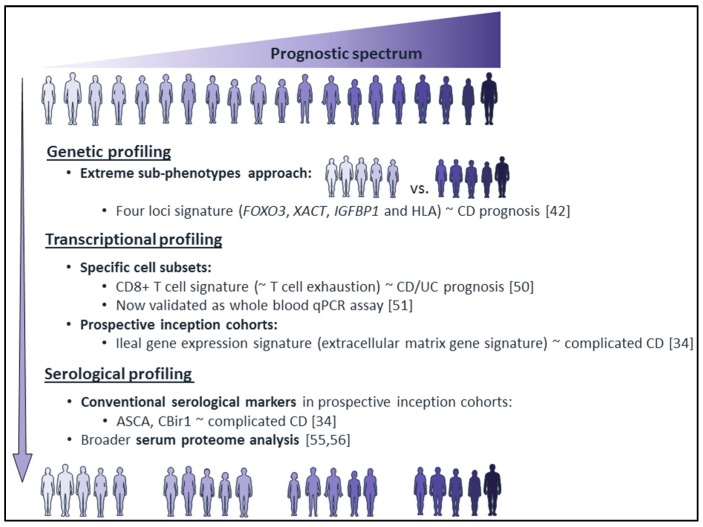
Prediction of IBD disease progression based on molecular profiling. Molecular profiling of disease prognosis using genetics, transcriptomics, and serology has been rapidly evolving. The most promising studies in the IBD filed and their general study designs are shown with references. CD: Crohn’s disease, UC: ulcerative colitis, ASCA: anti-*Saccharomyces cerevisiae* antibodies, Cbir1: anti-flagellin antibodies.

The clinical predictors of a poor outcome in CD have been identified as a young age (<40 years) at diagnosis, extensive small bowel disease, perianal fistula, smoking, and the need for corticosteroids at diagnosis [33]. In a prospective inception cohort study in pediatric CD patients with a new diagnosis—the RISK cohort [34], anti-*Saccharomyces cerevisiae* antibodies (ASCA) and anti-flagellin antibodies (CBir1) seropositivity were associated with complicated disease. Although the patients with these features have a significant risk of more complicated disease behavior, these clinical and serological factors have not led to optimal disease stratification at the time of diagnosis. Thus, the search for molecular markers to further predict IBD prognosis has been actively pursued (Figure 2).

### 3.2. Genetic Profiling

Predicting the course of IBD is important for optimizing treatments. Since genetic factors remain stable over time, are present long before disease onset, and are not open to subjective interpretation, they are promising candidates for disease prediction. However, only few associations between genetic variants and IBD phenotypes have been reported.

The most studied SNPs in the context of IBD phenotypes are the three well-known *NOD2* variants, particularly in CD. These variants have been associated with a faster onset of stricturing disease and the need for surgery [35,36,37,38,39]. However, these associations are not with prognosis per se, but are a secondary phenomenon driven by the association of *NOD2* variants with a younger age at diagnosis and ileal disease location [25]. Other retrospective cohort studies identified associations between known susceptibility variants and IBD phenotypes, but they often lack confirmation in other (prospective) cohorts [40,41]. There are criticisms that the tested SNPs are known susceptibility markers and may be less useful in differentiating patients (disease susceptibility loci versus disease-modifying loci). Some studies have, however, looked beyond the established susceptibility variants. Alonso et al. conducted a GWAS to identify genetic markers for 17 clinical phenotypes of CD (on the basis of disease location, behavior, course, age at onset, and extraintestinal manifestations) and found an association between *MAGI1* rs11924265 and complex stricturing disease [38]. The largest international study to date evaluating genotype–phenotype associations in IBD only found a few genome-wide significant associations (*NOD2*, *MHC*, and *MST1* 3p21) with age at onset, disease location, or behavior as defined by the Montreal classification, with the latter mostly driven by associations with disease location [25].

A more effective approach could be to utilize extreme sub-phenotypes, i.e., patients at opposite ends of the prognostic spectrum (Figure 2). By comparing contrasting clinical courses, Lee et al. identified four loci that are associated with CD prognosis: *FOXO3*, *XACT*, a region upstream of *IGFBP1*, and the HLA region [42]. None of these four loci were associated with disease susceptibility. Visschedijk et al. performed a within-case analysis by comparing extreme phenotypes with a strict definition for fibrostenotic disease (i.e., two or more resections due to confirmed ileal stenosis) [43]. SNP rs11861007 (located in *WWOX* and in lncRNA RP11-679B19.1) was identified as a disease-modifying genetic variant associated with recurrent fibrostenotic CD. Using a similar approach, Kopylov et al. found that none of their studied susceptibility loci were associated with benign UC [44]. In East Asians with UC, the SNP located between HLA-DRA and HLA-DRB (rs9268877) was found to be associated with a poor prognosis but not with susceptibility [45]. These studies highlight the need for detailed clinically relevant sub-phenotypes and categorization by relevant clinical outcomes in IBD.

As for predicting overall IBD disease risk, linking polygenic risk scores with clinical outcomes may yield more meaningful results. Although IBD patients tend to have larger average PRS than non-IBD individuals, this has not produced substantial improvements in predicting their clinical course. Indeed, no associations could be shown between genetic burden and development of complicated disease or need for surgery [39,42,44,45,46,47].

Across different studies, the definition of phenotypes is often not the same. This is an important challenge in conducting genotype–phenotype studies and might be one of the reasons for the lack of replication for some of the found associations. It should also be noted that, because of incomplete penetrance and the complex genetic nature, the genotype is not fully predictive of the phenotype. Genetic markers will thus not be able to entirely predict the natural course of the disease or the clinical outcome. The role of other factors and markers, like transcriptomic or serological markers, should also be considered.

### 3.3. Transcriptional Profiling

With the successes seen in the oncology field, it was hoped that we would be able to use transcriptional signatures to predict disease prognosis in IBD. Gene expression signatures indeed are used in the prediction of cancer behavior, such as metastasis and response to chemotherapy [48,49]. Studies trying to link gene expression with disease progression in IBD have been less successful and somewhat disappointing. They often lack consistent replication and have typically been compromised by confounding factors such as clinical heterogeneity (e.g., medication use, disease severity).

Some more recent studies with smart study design seem to be starting to turn the tide (Figure 2). One of the most promising studies came from the UK. Researchers found a transcriptional signature in separated CD8^+^ T cells that was predictive of prognosis in CD and UC patients [50]. They could validate this signature by a whole-blood qPCR assay, allowing easier translation to the bedside, in a prospectively collected cohort of newly diagnosed UC and CD patients [51]. A prospective biomarker-stratified trial in the UK is currently investigating the feasibility of personalized CD therapy [52].

The last years, several groups have also invested in assembling prospective inception cohort studies of newly diagnosed patients naive to any disease-related therapies, hereby excluding possible confounding effects of medication. Kugathasan et al. gathered the largest prospective inception cohort of pediatric patients with CD to date (n = 913)—the RISK cohort [34]. They found that an upregulated ileal extracellular matrix gene signature at diagnosis is associated with a stricturing disease risk and presented a risk stratification model for complicated disease behavior based on clinical, serological, gene expression, and microbial factors defined at CD diagnosis [34] (Figure 2). Furthermore, transcriptional risk scores integrating GWAS and expression quantitative trait locus data (the effects of genetic variation on gene expression) with ileal gene expression in this same CD cohort could be used to distinguish a complicated clinical course (B2/B3) of CD [53]. The mucosal transcriptomes from treatment-naïve UC patients in the PROTECT cohort in turn revealed mitochondrial dysfunction in active UC and higher anti-inflammatory ALOX15 expression during remission [54]. These findings highlight the potential of transcriptome analysis as a guide to precision medicine.

### 3.4. Serological Profiling

The conventional serologic markers studied in the context of IBD are antibodies against bacterial antigens, such as ASCA and CBir1, and antibodies against neutrophil antigens (pANCA), as also described above for the RISK cohort. Recently, a broader assessment of differentially expressed inflammatory proteins by serum proteome analysis was performed mostly using the Olink multiplex panels (Olink Proteomics, Uppsala, Sweden). The IBD Character biomarker discovery initiative used these panels on their large set of prospectively included newly diagnosed treatment-naïve IBD patients and found a protein signature with significant association with treatment escalation [55,56]. Further study will need to validate these findings and show the clinical role of the identified signature and serum proteome analysis in the prediction of clinical sub-phenotypes.

## 4. The Predictability of Treatment Response

### 4.1. Case 4: 25-Year-Old Male with a Two-Year History of Ileocolonic CD

*Case description:* A 25-year-old male with a two-year history of ileocolonic CD had maintained clinical remission for two years with azathioprine. Three weeks before the visit, he developed cramping abdominal pain, nausea, vomiting, and diarrhea. His leukocyte count was 6500/μL, C-reactive protein (CRP) was 1.5 mg/dL (<0.6), and Crohn’s disease activity index (CDAI) was 350. He had smoked half a pack of cigarettes per day for five years. A CT enterograph revealed mucosal thickening, enhancement of the terminal ileum, and focal narrowing of the distal ileum without proximal dilatation. A colonoscopy revealed active longitudinal ulcers in the terminal ileum with multiple scars. Infliximab (5 mg/kg) was initiated with daily azathioprine. Endoscopic biopsies and blood sampling were conducted before the first infusion of infliximab. Two weeks later, his CDAI had decreased to 120, and maintenance with infliximab was planned. Will he have a well-sustained response to infliximab?

Between 20% and 30% of IBD patients are refractory to any given medication, despite optimal dose and duration. Besides response, treatment side effects and toxicity are also variable. The need to predict the response to therapy is as pressing as the need to predict the disease course in IBD and will become even more important as more classes of therapeutics become available (e.g., anti-TNF agents, cell adhesion molecule inhibitors, anti-IL-12/23 agents, and other small molecules). Some clinical factors (e.g., concurrent use of immunomodulators, age, smoking, disease duration and location) have been identified but were not sufficiently reliable for predicting response to anti-TNF agents [34,57,58,59]. To date, also no clear associations exist to justify the use of serological markers in the prediction of the response to (anti-TNF) therapy in clinical management [60,61,62].

### 4.2. Genetic Profiling

The question of whether genetics can help predict the response to IBD therapy has been at the forefront of many research efforts. Unfortunately, genetic markers have had limited success in predicting the outcomes of IBD therapy, again in contrast to other fields such as oncology, where molecular markers have shown clinical utility in predicting the response to chemotherapy. One of several examples in cancer treatment is cetuximab, for which the beneficial effects seem limited to patients with *KRAS* wild-type metastatic colorectal cancer [63].

Most pharmacogenetic studies on the response to biologics in IBD patients have considered single genes (*TNF*, *NOD2*, *FCGR3A*, *IL-23R, FcRn*) [62,64,65,66,67,68,69] or gene groups (e.g., apoptosis genes) [70] in smaller-size cohorts. Findings on these candidate gene studies, however, were largely inconsistent and could not be confirmed in larger cohorts. A recent systematic review and meta-analysis of 15 such studies reported associations of *TLR2*, *TLR4*, *TLR9*, *TNFRSF1A*, *IFNG*, *IL6*, and *IL1B* with response to infliximab in IBD [71]; however, the associations were relatively weak. Billiet et al. employed a PRS based on 140 validated CD risk loci [72]; however, this score did not affect an individual patient’s response to anti-TNF therapy [59]. Dubinsky et al. conducted an unbiased genome-wide association study in pediatric IBD patients to find novel ‘pharmacogenetic’ loci and a primary non-response to anti-TNFα [73]. Three loci were significant in a final predictive model: *TACR1* (Tachykanin Receptor 1), a receptor for substance P and known pro-inflammatory molecule, *PHACTR3* (Phosphatase And Actin Regulator 3) which is associated with the nuclear scaffold in proliferating cells, and *FAM19A4* (Family With Sequence Similarity 19 Member A4, C-C Motif Chemokine Like) which functions as a chemokine and regulator of immune cells in the brain. Another recent study used immunochip genotypes to identify genetic factors associated with response to anti-TNF therapy in CD patients and summed these factors into a genetic risk score [74]. They identified different genetic factors associated with non-response and with durable response, respectively, suggesting that these two response outcomes have distinct underlying mechanisms. The genetic associations identified in these studies require further independent confirmation. These findings possibly may not have any immediate clinical impact but they will increase our understanding of the complex mode of action of anti-TNF agents in CD.

Similar to sub-phenotypes of disease, treatment response is often defined subjectively and different across studies. Another factor that is important in the response to anti-TNF agents and other biologics is immunogenicity. An advantage is that immunogenicity is more objectively defined, and thus genetic markers for the formation of antibodies to anti-TNF drugs might be a more feasible target. The incidence of antibody formation against anti-TNF agents is estimated to range from 1% to 14% depending on the specific drug [75]. Many factors could influence antibody formation [76]: patient factors (age, genetic background), intrinsic drug factors (formulation, structure, stability), and extrinsic drug factors (method of administration, dosage, frequency, length of treatment). In multiple sclerosis, an association between HLA-DRB1 and the formation of antibodies towards interferon-β therapy is seen [77]. HLA-DRB1 alleles have also recently been associated with immunogenicity to infliximab in IBD patients [78]: the presence of arginine at position 74 and the absence of glutamate at position 71 in the peptide-binding groove of the HLA-DRB1 complex are associated with antibodies against infliximab. A recent prospective and observational UK-wide study, however, reported that it is HLA-DQA1*05 that is associated with antibody responses to infliximab and adalimumab and that immunogenicity in carrier patients is attenuated by concomitant immunomodulators [79]. Patients could therefore be genetically screened before initiating a treatment. However, it is unknown whether the carriage of this variant is also associated with a loss of response and not just with immunogenicity.

### 4.3. Transcriptional Profiling

Genome-wide mucosal gene expression studies have identified several candidate genes that are possibly predictive of response to a range of biologics. Arijs et al. reported mucosal gene expression signatures that are predictive of a (non-)response in infliximab-naive CD and UC patients [80,81]. Genes that showed a lower expression at baseline in responders than in non-responders were mainly involved in immune signaling. This suggests a potentially larger immune burden at baseline in non-responders to anti-TNF therapy. The large overlap of the predictive genes in UC and CD patients implies a shared mechanism of nonresponse to infliximab in both disorders. One of the key biomarkers was *IL13RA2* (IL-13 Receptor alpha 2), which was independently confirmed in adalimumab-treated CD patients [82]. In an *il13ra2* knock-out mouse model exposed to dextran sodium sulfate (DSS), il13ra2 on epithelial cells contributed to IBD by negatively influencing goblet cell responses and epithelial regeneration after intestinal damage [83]. This molecule was also found to drive fibrosis [84]. Notably, the gene expression signature identified by Arijs et al. was replicated in a phase 2a open-label study of 103 golimumab-treated patients with moderate-to-severe UC [85]. However, the prediction specificity for mucosal healing in that study was only 34%, thus limiting its clinical utility [85].

West et al. reported that elevated expression of the IL-6 family member oncostatin M (*OSM*) in the pre-treatment intestine correlated with failure of anti-TNF therapy, consistent with an increased OSM expression in inflamed tissue correlating with disease activity [86]. OSM drives pro-inflammatory cytokine production in the gut, ultimately inducing the infiltration of neutrophils, monocytes, and T cells. Finally, a recent extensive study by Schmitt et al. on the molecular mechanisms underlying anti-TNF therapy resistance reported a potential role of IL-23 in mediating this non-response, with an expansion of apoptosis-resistant TNFR2^+^IL23^+^ T cells associated with anti-TNF resistance in CD patients [87]. These authors additionally demonstrated a significant upregulation of mucosal IL-23p19, IL23R, and IL-17A during anti-TNF therapy in anti-TNF-refractory patients and identified IL23 as a suitable target in anti-TNF non-responders.

Whereas the previously mentioned studies have screened for markers at the mucosal side, Gaujoux et al. identified *TREM-1* downregulation in whole blood at baseline as predictive of anti-TNF non-response with an area under the curve of 94% [88]. However, the opposite has also been reported, with significant downregulation of *TREM-1* in whole blood, both at protein and mRNA level, at baseline in patients who achieved mucosal healing after anti-TNF therapy [89,90].

With new agents having entered the market, similar transcriptome studies examining differences between responsive and resistant IBD patients have been performed for these other agents as well. For etrolizumab, a humanized monoclonal antibody against the β7 integrin subunit designed to target α4β7 and αEβ7 integrin-expressing immune cells, data from a randomized placebo-controlled trial have been used. Patients with high integrin αE (*ITGAE*) and granzyme A (*GZMA*) gene expression in the pre-treatment colonic biopsy sample achieved better clinical remission with etrolizumab [91,92]. However, whether these biomarkers hold their promise in phase III programs and in larger prospective studies remains unknown. Histologic and gene expression changes before and after treatment with vedolizumab, a monoclonal antibody against the α4β7 integrin, were assessed in 41 UC patients from the GEMINI I and GEMINI long-term safety study [93]. Despite achieving mucosal healing, persistent histological and immune-related gene dysregulations remained in a portion of patients receiving vedolizumab treatment. Among the anti-TNF-naive UC patients who were refractory to standard therapy, a higher expression of *ADGRL2* (also called *LPHN2*) or *FGF7* was indicative of better candidates for vedolizumab than anti-TNF treatment.

Attempts to identify molecular markers for the response to biological therapy in IBD thus have achieved only limited success, partly because of confounding factors and the lack of uniformly defined objective response criteria. Treatment responses in a heterogeneous disorder such as IBD are influenced by many factors, including disease duration, behavior, severity, and inter-individual variations in drug pharmacokinetics. To advance the pharmacogenetic field, trials involving patient cohorts treated with fixed drug doses as well as well-defined and unified response criteria are necessary. Hence, multi-layered data from a standardized randomized controlled trial could provide a great opportunity to identify molecular markers with clinical applicability. Notably, a previous genome-wide pharmacogenetic study in asthma used available randomized controlled trials and identified and confirmed novel pharmacogenetic determinants in the *GLCCI1* gene that predicted the response to inhaled glucocorticoids [94]. Although several challenges remain in terms of robust reproducibility and clinical utility, the findings highlighted above emphasize that genomic and transcriptomic profiling before starting a treatment might help in identifying biomarker signatures and thus patients who are most likely to benefit from specific therapies.

## 5. Predictability of Adverse Events

### 5.1. Case 5: 28-Year-Old Female with a Three-Year History of UC

*Case description:* A 28-year-old female immigrant from South Korea with a three-year history of UC displayed inflammation confined to the rectum. Sulfasalazine and a mesalazine suppository were administered daily. She had experienced two flare-ups and progressed proximally to the splenic flexure in the past year, and azathioprine was planned with the tapering of prednisone. Do we need to perform genetic testing before administering any of these medications to avoid adverse events?

Adverse events related to IBD medications are typically rare but potentially life-threatening. Recent studies have reported that some rare complications of these therapies are associated with clinically useful genetic variations.

### 5.2. Thiopurines

Thiopurine analogues are the only class of drugs for which genetic testing is recommended and useful for IBD. Thiopurine-induced myelotoxicity is mainly explained by the complex metabolism of these agents, which results in the accumulation of potentially toxic metabolites (high concentrations of 6TGN or 6-thio-GTP/6-thio-dGTP). Azathioprine is metabolized by the enzymes TPMT (thiopurine methyl transferase) and NUDT15 (hydrolase/8-oxo-7,8-dihydrodeoxyguanosine triphosphate pyrophosphatase), whose activities are dependent on variation in their genes [95,96,97]. Thiopurines should be avoided in patients with a TPMT deficiency, a homozygous *NUDT15* variant genotype, or a *NUDT15*/*TPMT* variant diplotype. Moreover, hematologic toxicity can develop in patients who do not harbor a known variant of either *TPMT* or *NUDT15* and in those with normal TPMT activity levels. Genetic testing will not obviate the requirement for regularly monitoring blood counts and liver transaminase activity for the duration of thiopurine administration.

In practice, both genotyping for the most common *TPMT* variants and measuring TPMT enzyme activity can be performed. Both techniques have advantages, and the choice of test is partly dependent on availability. *TPMT* genotyping is easier to perform, but genotypes do not fully correlate with enzyme activity, particularly in wild-type (some patients will have reduced TPMT activity) or heterozygous (some will have normal TPMT activity) individuals [98,99,100,101]. Direct measurements can therefore more accurately identify cases with high TPMT activity that will metabolize 6-mercaptopurine (6-MP) to 6-methyl-MP and thus be resistant to thiopurine therapies. When *TPMT* genetic testing is performed, normal doses of azathioprine or 6-MP (2.5 and 1.5 mg/kg, respectively) can be administered to patients with a wild-type genotype or normal enzyme activity levels. When TPMT activity is intermediate or when patients are heterozygous for common *TPMT* variants, a dose reduction of 50% is recommended. Finally, patients with low or absent TPMT activity and/or harboring a compound heterozygous/homozygous mutation in the *TPMT* gene should not be given azathioprine or 6-MP because of a high risk of myelotoxicity.

Specifically, for the case described here, genotyping or activity assessment of this enzyme will play only a limited role in the prevention of thiopurine-induced leukopenia, as TPMT deficiencies are relatively rare among Asians. Notably, however, *NUDT15* coding variants (mainly p.R139C) are strongly associated with leukopenia with a high sensitivity and specificity in people of Asian ethnicity [96], independent of 6-TGN levels [102,103]. Because NUDT15 converts TGTP to TGMP (and TdGTP to TdGMP), it prevents the incorporation of these thiopurine metabolites into DNA (DNA-TG) [97]. *NUTD15* variations cause a loss of nucleotide diphosphatase activity, leading to excessive levels of thiopurine active metabolites (6-thio-GTP and/or 6-thio-dGTP) and toxicity [97]. Although NUDT15 variants are less common than TPMT in Caucasian populations, their effects were found to be substantial even in heterozygotes [104].

Another serious adverse event of thiopurine exposure is pancreatitis, which occurs in 4% of cases. A recent international GWAS by Heap et al. identified the HLA-DQA1*02:01-HLA-DRB1*07:01 haplotype to be associated with thiopurine-induced pancreatitis [105]. IBD patients who are heterozygous for rs2647087 have a 5–9% risk of this, whereas homozygotes have a 15–17% risk [105,106]. Although these latter variants are not yet used in the clinic, their inclusion in testing might substantially reduce the risk of serious adverse events related to thiopurine analogues.

### 5.3. 5-Aminosalicylate (5-ASA)

Nephrotoxicity is a rare idiosyncratic reaction to 5-ASA administration in IBD patients. A cohort of 151 such cases (5 definite and 146 probable cases) was recruited from 89 centers over a two-year period and analyzed by GWAS. Heap et al. identified an association of 5-ASA induced nephrotoxicity with the rs3135356 variant in the HLA region [107]. This signal was significantly strengthened above the GWS threshold when testing only biopsy-positive cases (OR = 3.11). The clinical benefits of this pre-genotyping remain limited, given the rarity and relatively modest effects of nephrotoxicity.

### 5.4. Anti-TNF Therapy

One of the most common adverse events associated with anti-TNF therapy are skin lesions. These lesions affect up to 25% of IBD patients receiving anti-TNF therapy [108]. Tillack et al. reported that a small group (n = 7) of IBD patients who had developed severe psoriasiform skin lesions and/or alopecia during anti-TNF therapy were effectively treated with ustekinumab, an anti-IL12/IL23 antibody. All seven patients were G/G wild-type carriers for the *IL23R* coding variant rs11209026 [109]. The wild-type status of this SNP is associated with increased T-helper 17 cytokine production, whereas carrying the minor allele implies a decreased secretion of this cytokine. Therefore, anti-IL23 treatment is efficacious in wild-type patients. Given the previously shown efficacy of anti-IL12/IL23 antibody therapy in CD, ustekinumab may be a suitable alternative to anti-TNF therapy in CD patients with anti-TNF-associated skin lesions. *IL23R* variant typing might contribute to treatment decisions in this case.

## 6. Conclusions

**Figure 3:**
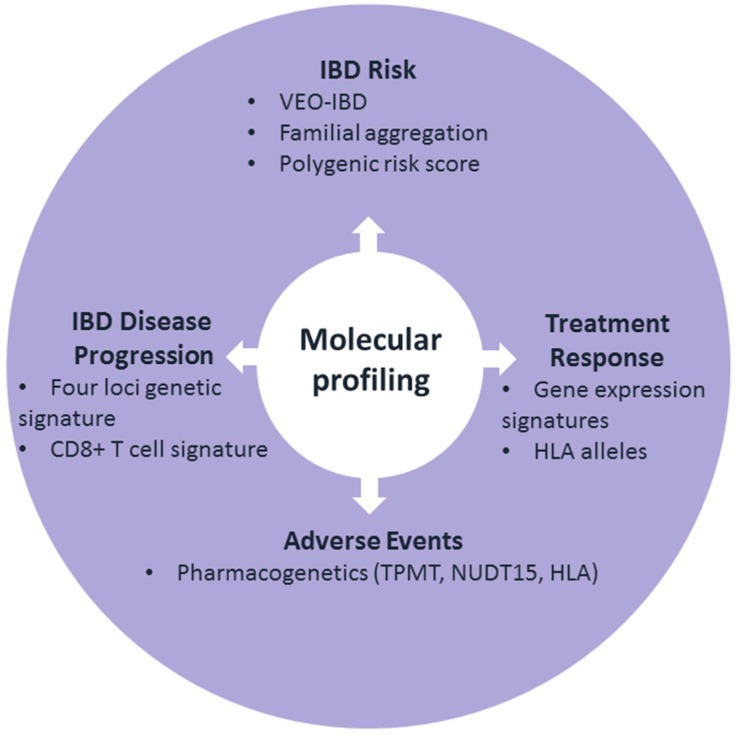
Schematic diagram of the currently most promising use of molecular profiling in inflammatory bowel disease. Some further details can be found in Table 2. VEO-IBD: very early onset IBD.

Although previous achievements in IBD genetics have provided a better insight into disease pathogenesis, many clinical application questions remain to be elucidated. In this review, we outlined current progress in molecular profiling for the prediction of IBD (subtypes) as well as some pointers to help answer questions from patients’ cases (see Figure 3 and Table 2 for an overview). Whereas clinical IBD classifications have focused on disease location, behavior, and age at onset, this classification does not correlate well with the natural disease course. The search for molecular markers to further define and predict IBD outcomes has moved forward rapidly with the help of modern technologies. The past decade has seen substantial successes in the use of GWAS to identify genetic variants of IBD susceptibility. However, molecular profiling of the clinical course and treatment responses in these patients lags far behind this susceptibility analysis. Clinical applications of molecular profiling do exist in other disease fields. In cancer management, targeted therapy based on genetic data is widely applied (e.g., cetuximab for KRAS wild-type colorectal cancer, dabrafenib for cancers with V600E BRAF mutation). In cardiovascular disease, pharmacogenetic profiling for clopidogrel, warfarin, acenocoumarol, and simvastatin dosing is recommended [110,111]. At the same time, there are some obstacles that need to be overcome to fully implement pharmacogenetic testing in the clinical practice: we need robust evidence for cost effectiveness, physicians and patients need to be aware and trained, and commercial pharmacogenetic panels need to be developed [111]. For IBD, to date, pharmacogenetic research has also produced the most success in the context of molecular patient profiling (e.g., *TPMT* and *NUDT15* genetic testing in the context of thiopurine-induced myelotoxicity, Table 2).

Prediction of the clinical course based on molecular characterization of IBD is influenced by many elements including clinical, environmental, and microbiome factors, and thus this research field has produced only modest successes to date. The future direction of this research field likely involves expanding upon existing gene expression or genetic profiling to include multiple omics and thus integrate the different omic layers, i.e., clinical, serological, (epi-)genetic, and microbial factors. This was exemplified to some extent by the RISK cohort findings [34]. Innovative efforts at integrating different data sets could provide important new insights into risk identification and optimized treatment for IBD in the near future [112]. Another important direction is to characterize IBD disease progression and response to treatment at an in-depth cellular level for a deeper definition of the pathophysiological context. Although not without merit, gene expression analysis of bulk tissues has also important intrinsic limitations, as it uses the averaged gene expression of the bulk population of cells, and hence, any transcriptional variation detected will predominantly reflect differences in the cellular composition between samples. Recent advances in biotechnology now allow gene expression measurements in individual cells (single-cell RNA-seq) and thus the identification and quantification of cells based on their intrinsic transcriptome. A few studies have been published so far [113,114,115,116]. For example, the study by Martin et al. used high-resolution single-cell mapping of inflammatory lesions in patients with CD to characterize the ileal lamina propria cellular landscape, hereby revealing a (complex) cellular signature of anti-TNF resistance [113]. Further studies are warranted, incorporating smaller scale single-cell data with bulk RNA-seq data. Taken together, the requirements for pharmacogenetic and other predictive studies to advance this field include: (i) detailed clinical, genomic, and follow-up data; (ii) standardized drug dose levels and fixed endpoints and criteria for treatment responses; (iii) analyzing data integrated from multiple “omes”; (iv) applying the newest technologies; (v) a culture of collaboration and data sharing. A clinical trial setting may be the optimal and preferred method to achieve these requirements, and longitudinal collections of samples from these patients should be emphasized as an absolute requirement.

## Figures and Tables

**Table 1:** Risk factors for inflammatory bowel disease (IBD).

	Relative Risk	Absolute Risk
Lifetime risk for IBD	1×	1.3%
Familial aggregation		
IBD in first-degree relatives	4–15×	5.2–19.5%
Both parents affected	20–25×	30%
Genetic factors		
*NOD2* variant ^1^	2.1–3.0×	2.7–3.9%
Typical susceptibility variants	1.1–1.5×	1.4–2.0%
PRS—Individuals in the top 1%	3.9×	5.1%
Environmental factor		
Current smoking ^1^	1.8×	2.3%

PRS, polygenic risk score. ^1^ Risk factor for Crohn’s disease.

**Table 2:** Current status of the prediction of several aspects of inflammatory bowel disease based on molecular profiling.

Category	Application	Usability ^1^
Risk/diagnosis	VEO-IBD: application of (targeted) next-generation sequencing	✔
	Familial IBD: risk of IBD is increased 4–15-fold in first-degree relatives of patients	✔
	Sporadic/familial IBD: individual risk variants	✘
	Sporadic/familial IBD: polygenic risk scores	✔
Disease progression	Genetic testing: individual susceptibility variants	✘
	Genetic testing: polygenic risk scores	✘
	Genetic testing: variants from extreme sub-phenotype approaches [42,43,45]	✔
	Transcriptional profiling: CD8+ T cell transcription signature [50,51]	✔
Treatment response	Genetic testing: polygenic risk scores	✘
	Genetic testing: Immunogenicity (HLA-DQA1*05) [79]	✔
	Transcriptional profiling: *OSM*, *TREM1*, gene signature including *IL13RA2* [80,81,82,86,88,89,90]	✔
Adverse events	Thiopurine-induced myelotoxicity: *TPMT*, *NUDT15* genetic testing	✔
	Thiopurine-induced pancreatitis: HLA genetic testing [105,106]	✔
	Skin lesions under anti-TNF therapy: *IL23R* rs11209026 genetic testing [109]	✔

^1^ Green: established; blue: promising/clinical trial ongoing; orange: promising/replication needed; red: currently unaccepted as predictors for the indicated category.

## References

[1] Silverberg M.S., Satsangi J., Ahmad T., Arnott I.D., Bernstein C.N., Brant S.R., Caprilli R., Colombel J.F., Gasche C., Geboes K. (2005). Toward an integrated clinical, molecular and serological classification of inflammatory bowel disease: Report of a Working Party of the 2005 Montreal World Congress of Gastroenterology. Can. J. Gastroenterol..

[2] Satsangi J., Silverberg M.S., Vermeire S., Colombel J.F. (2006). The Montreal classification of inflammatory bowel disease: Controversies, consensus, and implications. Gut.

[3] Spekhorst L.M., Visschedijk M.C., Alberts R., Festen E.A., van der Wouden E.J., Dijkstra G., Weersma R.K. (2014). Performance of the Montreal classification for inflammatory bowel diseases. World J. Gastroenterol..

[4] Liu J.Z., van Sommeren S., Huang H., Ng S.C., Alberts R., Takahashi A., Ripke S., Lee J.C., Jostins L., Shah T. (2015). Association analyses identify 38 susceptibility loci for inflammatory bowel disease and highlight shared genetic risk across populations. Nat. Genet..

[5] de Lange K.M., Moutsianas L., Lee J.C., Lamb C.A., Luo Y., Kennedy N.A., Jostins L., Rice D.L., Gutierrez-Achury J., Ji S.G. (2017). Genome-wide association study implicates immune activation of multiple integrin genes in inflammatory bowel disease. Nat. Genet..

[6] Mirkov M.U., Verstockt B., Cleynen I. (2017). Genetics of inflammatory bowel disease: Beyond NOD2. Lancet Gastroenterol. Hepatol..

[7] Ananthakrishnan A.N. (2015). Epidemiology and risk factors for IBD. Nat. Rev. Gastroenterol. Hepatol..

[8] Ng S.C., Tang W., Ching J.Y., Wong M., Chow C.M., Hui A.J., Wong T.C., Leung V.K., Tsang S.W., Yu H.H. (2013). Incidence and phenotype of inflammatory bowel disease based on results from the Asia-pacific Crohn’s and colitis epidemiology study. Gastroenterology.

[9] M’Koma A.E. (2013). Inflammatory bowel disease: An expanding global health problem. Clin. Med. Insights Gastroenterol..

[10] Xu F., Dahlhamer J.M., Zammitti E.P., Wheaton A.G., Croft J.B. (2018). Health-Risk Behaviors and Chronic Conditions Among Adults with Inflammatory Bowel Disease - United States, 2015 and 2016. MMWR Morb. Mortal. Wkly. Rep..

[11] Orholm M., Munkholm P., Langholz E., Nielsen O.H., Sorensen T.I., Binder V. (1991). Familial occurrence of inflammatory bowel disease. N. Engl. J. Med..

[12] Halme L., Paavola-Sakki P., Turunen U., Lappalainen M., Farkkila M., Kontula K. (2006). Family and twin studies in inflammatory bowel disease. World J. Gastroenterol..

[13] Park J.B., Yang S.K., Byeon J.S., Park E.R., Moon G., Myung S.J., Park W.K., Yoon S.G., Kim H.S., Lee J.G. (2006). Familial occurrence of inflammatory bowel disease in Korea. Inflamm. Bowel. Dis..

[14] Moller F.T., Andersen V., Wohlfahrt J., Jess T. (2015). Familial risk of inflammatory bowel disease: A population-based cohort study 1977-2011. Am. J. Gastroenterol..

[15] Bennett R.A., Rubin P.H., Present D.H. (1991). Frequency of inflammatory bowel disease in offspring of couples both presenting with inflammatory bowel disease. Gastroenterology.

[16] Laharie D., Debeugny S., Peeters M., Van Gossum A., Gower-Rousseau C., Belaiche J., Fiasse R., Dupas J.L., Lerebours E., Piotte S. (2001). Inflammatory bowel disease in spouses and their offspring. Gastroenterology.

[17] Henriksen M., Jahnsen J., Lygren I., Vatn M.H., Moum B. (2007). Are there any differences in phenotype or disease course between familial and sporadic cases of inflammatory bowel disease? Results of a population-based follow-up study. Am. J. Gastroenterol..

[18] Joossens M., Van Steen K., Branche J., Sendid B., Rutgeerts P., Vasseur F., Poulain D., Broly F., Colombel J.F., Vermeire S. (2010). Familial aggregation and antimicrobial response dose-dependently affect the risk for Crohn’s disease. Inflamm. Bowel Dis..

[19] Mahid S.S., Minor K.S., Soto R.E., Hornung C.A., Galandiuk S. (2006). Smoking and inflammatory bowel disease: A meta-analysis. Mayo Clin. Proc..

[20] Khera A.V., Chaffin M., Aragam K.G., Haas M.E., Roselli C., Choi S.H., Natarajan P., Lander E.S., Lubitz S.A., Ellinor P.T. (2018). Genome-wide polygenic scores for common diseases identify individuals with risk equivalent to monogenic mutations. Nat. Genet..

[21] Weersma R.K., Stokkers P.C., Cleynen I., Wolfkamp S.C., Henckaerts L., Schreiber S., Dijkstra G., Franke A., Nolte I.M., Rutgeerts P. (2009). Confirmation of multiple Crohn’s disease susceptibility loci in a large Dutch-Belgian cohort. Am. J. Gastroenterol..

[22] Chen G.B., Lee S.H., Montgomery G.W., Wray N.R., Visscher P.M., Gearry R.B., Lawrance I.C., Andrews J.M., Bampton P., Mahy G. (2017). Performance of risk prediction for inflammatory bowel disease based on genotyping platform and genomic risk score method. BMC Med. Genet..

[23] Kevans D., Silverberg M.S., Borowski K., Griffiths A., Xu W., Onay V., Paterson A.D., Knight J., Croitoru K. (2016). IBD Genetic Risk Profile in Healthy First-Degree Relatives of Crohn’s Disease Patients. J. Crohns Colitis.

[24] Borren N.Z., Conway G., Garber J.J., Khalili H., Budree S., Mallick H., Yajnik V., Xavier R.J., Ananthakrishnan A.N. (2018). Differences in Clinical Course, Genetics, and the Microbiome Between Familial and Sporadic Inflammatory Bowel Diseases. J. Crohns Colitis.

[25] Cleynen I., Boucher G., Jostins L., Schumm L.P., Zeissig S., Ahmad T., Andersen V., Andrews J.M., Annese V., Brand S. (2016). Inherited determinants of Crohn’s disease and ulcerative colitis phenotypes: A genetic association study. Lancet.

[26] Uhlig H.H., Schwerd T., Koletzko S., Shah N., Kammermeier J., Elkadri A., Ouahed J., Wilson D.C., Travis S.P., Turner D. (2014). The diagnostic approach to monogenic very early onset inflammatory bowel disease. Gastroenterology.

[27] Uhlig H.H. (2013). Monogenic diseases associated with intestinal inflammation: Implications for the understanding of inflammatory bowel disease. Gut.

[28] Uhlig H.H., Schwerd T. (2016). From Genes to Mechanisms: The Expanding Spectrum of Monogenic Disorders Associated with Inflammatory Bowel Disease. Inflamm. Bowel Dis..

[29] Shouval D.S., Biswas A., Kang Y.H., Griffith A.E., Konnikova L., Mascanfroni I.D., Redhu N.S., Frei S.M., Field M., Doty A.L. (2016). Interleukin 1beta Mediates Intestinal Inflammation in Mice and Patients With Interleukin 10 Receptor Deficiency. Gastroenterology.

[30] Nijman I.J., van Montfrans J.M., Hoogstraat M., Boes M.L., van de Corput L., Renner E.D., van Zon P., van Lieshout S., Elferink M.G., van der Burg M. (2014). Targeted next-generation sequencing: A novel diagnostic tool for primary immunodeficiencies. J. Allergy Clin. Immunol..

[31] de Koning T.J., Jongbloed J.D., Sikkema-Raddatz B., Sinke R.J. (2015). Targeted next-generation sequencing panels for monogenetic disorders in clinical diagnostics: The opportunities and challenges. Expert Rev. Mol. Diagn..

[32] Fazeli W., Karakaya M., Herkenrath P., Vierzig A., Dotsch J., von Kleist-Retzow J.C., Cirak S. (2016). Mendeliome sequencing enables differential diagnosis and treatment of neonatal lactic acidosis. Mol. Cell. Pediatr..

[33] Beaugerie L., Sokol H. (2012). Clinical, serological and genetic predictors of inflammatory bowel disease course. World J. Gastroenterol..

[34] Kugathasan S., Denson L.A., Walters T.D., Kim M.O., Marigorta U.M., Schirmer M., Mondal K., Liu C., Griffiths A., Noe J.D. (2017). Prediction of complicated disease course for children newly diagnosed with Crohn’s disease: A multicentre inception cohort study. Lancet.

[35] Abreu M.T., Taylor K.D., Lin Y.C., Hang T., Gaiennie J., Landers C.J., Vasiliauskas E.A., Kam L.Y., Rojany M., Papadakis K.A. (2002). Mutations in NOD2 are associated with fibrostenosing disease in patients with Crohn’s disease. Gastroenterology.

[36] Ahmad T., Armuzzi A., Bunce M., Mulcahy-Hawes K., Marshall S.E., Orchard T.R., Crawshaw J., Large O., de Silva A., Cook J.T. (2002). The molecular classification of the clinical manifestations of Crohn’s disease. Gastroenterology.

[37] Seiderer J., Schnitzler F., Brand S., Staudinger T., Pfennig S., Herrmann K., Hofbauer K., Dambacher J., Tillack C., Sackmann M. (2006). Homozygosity for the CARD15 frameshift mutation 1007fs is predictive of early onset of Crohn’s disease with ileal stenosis, entero-enteral fistulas, and frequent need for surgical intervention with high risk of re-stenosis. Scand. J. Gastroenterol..

[38] Alonso A., Domènech E., Julià A., Panés J., García-Sánchez V., Mateu P.N., Gutiérrez A., Gomollón F., Mendoza J.L., Garcia-Planella E. (2015). Identification of Risk Loci for Crohn’s Disease Phenotypes Using a Genome-Wide Association Study. Gastroenterology.

[39] Cleynen I., Gonzalez J.R., Figueroa C., Franke A., McGovern D., Bortlik M., Crusius B.J., Vecchi M., Artieda M., Szczypiorska M. (2013). Genetic factors conferring an increased susceptibility to develop Crohn’s disease also influence disease phenotype: Results from the IBDchip European Project. Gut.

[40] Prescott N.J., Fisher S.A., Franke A., Hampe J., Onnie C.M., Soars D., Bagnall R., Mirza M.M., Sanderson J., Forbes A. (2007). A nonsynonymous SNP in ATG16L1 predisposes to ileal Crohn’s disease and is independent of CARD15 and IBD5. Gastroenterology.

[41] Henckaerts L., Van Steen K., Verstreken I., Cleynen I., Franke A., Schreiber S., Rutgeerts P., Vermeire S. (2009). Genetic risk profiling and prediction of disease course in Crohn’s disease patients. Clin. Gastroenterol. Hepatol..

[42] Lee J.C., Biasci D., Roberts R., Gearry R.B., Mansfield J.C., Ahmad T., Prescott N.J., Satsangi J., Wilson D.C., Jostins L. (2017). Genome-wide association study identifies distinct genetic contributions to prognosis and susceptibility in Crohn’s disease. Nat. Genet..

[43] Visschedijk M.C., Spekhorst L.M., Cheng S.C., van Loo E.S., Jansen B.D., Blokzijl T., Kil H., De Jong D.J., Pierik M., Maljaars J.P. (2018). Genomic and Expression Analyses Identify a Disease-Modifying Variant for Fibrostenotic Crohn’s Disease. J. Crohns Colitis.

[44] Kopylov U., Boucher G., Waterman M., Rivers C.R., Patel M., Cho J.H., Colombel J.F., Duerr R.H., Binion D., McGovern D.P. (2016). Genetic Predictors of Benign Course of Ulcerative Colitis-A North American Inflammatory Bowel Disease Genetics Consortium Study. Inflamm. Bowel Dis..

[45] Lee H.S., Yang S.K., Hong M., Jung S., Kim B.M., Moon J.W., Park S.H., Ye B.D., Oh S.H., Kim K.M. (2018). An intergenic variant rs9268877 between HLA-DRA and HLA-DRB contributes to the clinical course and long-term outcome of ulcerative colitis. J. Crohns Colitis.

[46] Jakobsen C., Cleynen I., Andersen P.S., Vermeire S., Munkholm P., Paerregaard A., Wewer V. (2014). Genetic susceptibility and genotype–phenotype association in 588 Danish children with inflammatory bowel disease. J. Crohns Colitis.

[47] Ananthakrishnan A.N., Huang H., Nguyen D.D., Sauk J., Yajnik V., Xavier R.J. (2014). Differential effect of genetic burden on disease phenotypes in Crohn’s disease and ulcerative colitis: Analysis of a North American cohort. Am. J. Gastroenterol..

[48] Aziz M.A., Yousef Z., Saleh A.M., Mohammad S., Al Knawy B. (2017). Towards personalized medicine of colorectal cancer. Crit. Rev. Oncol. Hematol..

[49] Reck M., Rabe K.F. (2017). Precision Diagnosis and Treatment for Advanced Non-Small-Cell Lung Cancer. N. Engl. J. Med..

[50] Lee J.C., Lyons P.A., McKinney E.F., Sowerby J.M., Carr E.J., Bredin F., Rickman H.M., Ratlamwala H., Hatton A., Rayner T.F. (2011). Gene expression profiling of CD8+ T cells predicts prognosis in patients with Crohn disease and ulcerative colitis. J. Clin. Investig..

[51] Biasci D., Lee J.C., Noor N.M., Pombal D.R., Hou M., Lewis N., Ahmad T., Hart A., Parkes M., McKinney E.F. (2019). A blood-based prognostic biomarker in IBD. Gut.

[52] Parkes M., Noor N.M., Dowling F., Leung H., Bond S., Whitehead L., Upponi S., Kinnon P., Sandham A.P., Lyons P.A. (2018). PRedicting Outcomes For Crohn’s dIsease using a moLecular biomarkEr (PROFILE): Protocol for a multicentre, randomised, biomarker-stratified trial. BMJ Open.

[53] Marigorta U.M., Denson L.A., Hyams J.S., Mondal K., Prince J., Walters T.D., Griffiths A., Noe J.D., Crandall W.V., Rosh J.R. (2017). Transcriptional risk scores link GWAS to eQTLs and predict complications in Crohn’s disease. Nat. Genet..

[54] Haberman Y., Karns R., Dexheimer P.J., Schirmer M., Somekh J., Jurickova I., Braun T., Novak E., Bauman L., Collins M.H. (2019). Ulcerative colitis mucosal transcriptomes reveal mitochondriopathy and personalized mechanisms underlying disease severity and treatment response. Nat. Commun..

[55] Kalla R., Kennedy N.A., Hjelm F.N., Modig E., Sundell M., Söderholm J., Andreassen B.K., Bergemalm D., Ventham N.T., Hjortswang H. (2016). DOP082 Proximity extension assay immunoassay technology identifies novel serum biomarkers that can diagnose and classify inflammatory bowel diseases: IBD Character Consortium. J. Crohns Colitis.

[56] Kalla R., Adams A., Vatn S., Bergemalm D., Ricanek P., Lindstrøm J., Ocklind A., Nordberg N., Kennedy N., Ventham N. (2017). OP022 Proximity extension assay based proteins show immune cell specificity and can diagnose and predict outcomes in inflammatory bowel diseases: IBD Character study. J. Crohns Colitis.

[57] Gonczi L., Vegh Z., Golovics P.A., Rutka M., Gecse K.B., Bor R., Farkas K., Szamosi T., Bene L., Gasztonyi B. (2017). Prediction of Short- and Medium-term Efficacy of Biosimilar Infliximab Therapy. Do Trough Levels and Antidrug Antibody Levels or Clinical And Biochemical Markers Play the More Important Role?. J. Crohns Colitis.

[58] Reinisch W., Colombel J.F., Sandborn W.J., Mantzaris G.J., Kornbluth A., Adedokun O.J., Miller M., Tang K.L., Rutgeerts P., Cornillie F. (2015). Factors associated with short- and long-term outcomes of therapy for Crohn’s disease. Clin. Gastroenterol. Hepatol..

[59] Billiet T., Papamichael K., de Bruyn M., Verstockt B., Cleynen I., Princen F., Singh S., Ferrante M., Van Assche G., Vermeire S. (2015). A Matrix-based Model Predicts Primary Response to Infliximab in Crohn’s Disease. J. Crohns Colitis.

[60] Arnott I.D., Landers C.J., Nimmo E.J., Drummond H.E., Smith B.K., Targan S.R., Satsangi J. (2004). Sero-reactivity to microbial components in Crohn’s disease is associated with disease severity and progression, but not NOD2/CARD15 genotype. Am. J. Gastroenterol..

[61] Esters N., Pierik M., van Steen K., Vermeire S., Claessens G., Joossens S., Vlietinck R., Rutgeerts P. (2004). Transmission of CARD15 (NOD2) variants within families of patients with inflammatory bowel disease. Am. J. Gastroenterol..

[62] Taylor K.D., Plevy S.E., Yang H., Landers C.J., Barry M.J., Rotter J.I., Targan S.R. (2001). ANCA pattern and LTA haplotype relationship to clinical responses to anti-TNF antibody treatment in Crohn’s disease. Gastroenterology.

[63] Van Cutsem E., Kohne C.H., Hitre E., Zaluski J., Chang Chien C.R., Makhson A., D’Haens G., Pinter T., Lim R., Bodoky G. (2009). Cetuximab and chemotherapy as initial treatment for metastatic colorectal cancer. N. Engl. J. Med..

[64] Mascheretti S., Hampe J., Kuhbacher T., Herfarth H., Krawczak M., Folsch U.R., Schreiber S. (2002). Pharmacogenetic investigation of the TNF/TNF-receptor system in patients with chronic active Crohn’s disease treated with infliximab. Pharmacogenom. J..

[65] Pierik M., Vermeire S., Steen K.V., Joossens S., Claessens G., Vlietinck R., Rutgeerts P. (2004). Tumour necrosis factor-alpha receptor 1 and 2 polymorphisms in inflammatory bowel disease and their association with response to infliximab. Aliment. Pharmacol. Ther..

[66] Billiet T., Dreesen E., Cleynen I., Wollants W.J., Ferrante M., Van Assche G., Gils A., Vermeire S. (2016). A Genetic Variation in the Neonatal Fc-Receptor Affects Anti-TNF Drug Concentrations in Inflammatory Bowel Disease. Am. J. Gastroenterol..

[67] Louis E.J., Watier H.E., Schreiber S., Hampe J., Taillard F., Olson A., Thorne N., Zhang H., Colombel J.F. (2006). Polymorphism in IgG Fc receptor gene FCGR3A and response to infliximab in Crohn’s disease: A subanalysis of the ACCENT I study. Pharm. Genom..

[68] Louis E., El Ghoul Z., Vermeire S., Dall’Ozzo S., Rutgeerts P., Paintaud G., Belaiche J., De Vos M., Van Gossum A., Colombel J.F. (2004). Association between polymorphism in IgG Fc receptor IIIa coding gene and biological response to infliximab in Crohn’s disease. Aliment Pharmacol. Ther..

[69] Vermeire S., Louis E., Rutgeerts P., De Vos M., Van Gossum A., Belaiche J., Pescatore P., Fiasse R., Pelckmans P., Vlietinck R. (2002). NOD2/CARD15 does not influence response to infliximab in Crohn’s disease. Gastroenterology.

[70] Hlavaty T., Pierik M., Henckaerts L., Ferrante M., Joossens S., van Schuerbeek N., Noman M., Rutgeerts P., Vermeire S. (2005). Polymorphisms in apoptosis genes predict response to infliximab therapy in luminal and fistulizing Crohn’s disease. Aliment Pharmacol. Ther..

[71] Bek S., Nielsen J.V., Bojesen A.B., Franke A., Bank S., Vogel U., Andersen V. (2016). Systematic review: Genetic biomarkers associated with anti-TNF treatment response in inflammatory bowel diseases. Aliment Pharmacol. Ther..

[72] Jostins L., Ripke S., Weersma R.K., Duerr R.H., McGovern D.P., Hui K.Y., Lee J.C., Schumm L.P., Sharma Y., Anderson C.A. (2012). Host-microbe interactions have shaped the genetic architecture of inflammatory bowel disease. Nature.

[73] Dubinsky M.C., Mei L., Friedman M., Dhere T., Haritunians T., Hakonarson H., Kim C., Glessner J., Targan S.R., McGovern D.P. (2010). Genome wide association (GWA) predictors of anti-TNFα therapeutic responsiveness in pediatric inflammatory bowel disease. Inflamm. Bowel Dis..

[74] Barber G.E., Yajnik V., Khalili H., Giallourakis C., Garber J., Xavier R., Ananthakrishnan A.N. (2016). Genetic Markers Predict Primary Non-Response and Durable Response To Anti-TNF Biologic Therapies in Crohn’s Disease. Am. J. Gastroenterol..

[75] Vande Casteele N., Gils A. (2015). Pharmacokinetics of anti-TNF monoclonal antibodies in inflammatory bowel disease: Adding value to current practice. J. Clin. Pharmacol..

[76] Dreesen E., Gils A., Vermeire S. (2018). Pharmacokinetic Modeling and Simulation of Biologicals in Inflammatory Bowel Disease: The Dawning of a New Era for Personalized Treatment. Curr. Drug Targets.

[77] Weber F., Cepok S., Wolf C., Berthele A., Uhr M., Bettecken T., Buck D., Hartung H.P., Holsboer F., Muller-Myhsok B. (2012). Single-nucleotide polymorphisms in HLA- and non-HLA genes associated with the development of antibodies to interferon-beta therapy in multiple sclerosis patients. Pharmacogenomics J..

[78] Billiet T., Vande Casteele N., Van Stappen T., Princen F., Singh S., Gils A., Ferrante M., Van Assche G., Cleynen I., Vermeire S. (2015). Immunogenicity to infliximab is associated with HLA-DRB1. Gut.

[79] Sazonovs A., Kennedy N.A., Bewshea C., Moutsianas L., Walker G.J., De Lange K., Goodhand J., Anderson C., Barrett J., Consortium P.I. (2018). OP013 HLA-DQA1 contributes to the development of antibodies to anti-TNF therapy in Crohn’s disease. J. Crohns Colitis.

[80] Arijs I., Li K., Toedter G., Quintens R., Van Lommel L., Van Steen K., Leemans P., De Hertogh G., Lemaire K., Ferrante M. (2009). Mucosal gene signatures to predict response to infliximab in patients with ulcerative colitis. Gut.

[81] Arijs I., Quintens R., Van Lommel L., Van Steen K., De Hertogh G., Lemaire K., Schraenen A., Perrier C., Van Assche G., Vermeire S. (2010). Predictive value of epithelial gene expression profiles for response to infliximab in Crohn’s disease. Inflamm. Bowel Dis..

[82] Verstockt B., Verstockt S., Creyns B., Tops S., Van Assche G., Gils A., Ceuppens J.L., Vermeire S., Ferrante M., Breynaert C. (2019). Mucosal IL13RA2 expression predicts nonresponse to anti-TNF therapy in Crohn’s disease. Aliment Pharmacol. Ther..

[83] Verstockt B., Perrier C., De Hertogh G., Cremer J., Creyns B., Van Assche G., Ferrante M., Ceuppens J.L., Vermeire S., Breynaert C. (2018). Effects of Epithelial IL-13Ralpha2 Expression in Inflammatory Bowel Disease. Front. Immunol..

[84] Strober W., Kitani A., Fichtner-Feigl S., Fuss I.J. (2009). The signaling function of the IL-13Ralpha2 receptor in the development of gastrointestinal fibrosis and cancer surveillance. Curr. Mol. Med..

[85] Telesco S.E., Brodmerkel C., Zhang H., Kim L.L., Johanns J., Mazumder A., Li K., Baribaud F., Curran M., Strauss R. (2018). Gene Expression Signature for Prediction of Golimumab Response in a Phase 2a Open-Label Trial of Patients With Ulcerative Colitis. Gastroenterology.

[86] West N.R., Hegazy A.N., Owens B.M.J., Bullers S.J., Linggi B., Buonocore S., Coccia M., Görtz D., This S., Stockenhuber K. (2017). Oncostatin M drives intestinal inflammation and predicts response to tumor necrosis factor-neutralizing therapy in patients with inflammatory bowel disease. Nat. Med..

[87] Schmitt H., Billmeier U., Dieterich W., Rath T., Sonnewald S., Reid S., Hirschmann S., Hildner K., Waldner M.J., Mudter J. (2019). Expansion of IL-23 receptor bearing TNFR2+ T cells is associated with molecular resistance to anti-TNF therapy in Crohn’s disease. Gut.

[88] Gaujoux R., Starosvetsky E., Maimon N., Vallania F., Bar-Yoseph H., Pressman S., Weisshof R., Goren I., Rabinowitz K., Waterman M. (2019). Cell-centred meta-analysis reveals baseline predictors of anti-TNFalpha non-response in biopsy and blood of patients with IBD. Gut.

[89] Verstockt B., Verstockt S., Blevi H., Cleynen I., de Bruyn M., Van Assche G., Vermeire S., Ferrante M. (2018). TREM-1, the ideal predictive biomarker for endoscopic healing in anti-TNF-treated Crohn’s disease patients?. Gut.

[90] Verstockt B., Verstockt S., Dehairs J., Ballet V., Blevi H., Wollants W.J., Breynaert C., Van Assche G., Vermeire S., Ferrante M. (2019). Low TREM1 expression in whole blood predicts anti-TNF response in inflammatory bowel disease. EBioMedicine.

[91] Tew G.W., Hackney J.A., Gibbons D., Lamb C.A., Luca D., Egen J.G., Diehl L., Eastham Anderson J., Vermeire S., Mansfield J.C. (2016). Association Between Response to Etrolizumab and Expression of Integrin αE and Granzyme A in Colon Biopsies of Patients With Ulcerative Colitis. Gastroenterology.

[92] Vermeire S., O’Byrne S., Keir M., Williams M., Lu T.T., Mansfield J.C., Lamb C.A., Feagan B.G., Panes J., Salas A. (2014). Etrolizumab as induction therapy for ulcerative colitis: A randomised, controlled, phase 2 trial. Lancet.

[93] Arijs I., De Hertogh G., Lemmens B., Van Lommel L., de Bruyn M., Vanhove W., Cleynen I., Machiels K., Ferrante M., Schuit F. (2018). Effect of vedolizumab (anti-alpha4beta7-integrin) therapy on histological healing and mucosal gene expression in patients with UC. Gut.

[94] Tantisira K.G., Lasky-Su J., Harada M., Murphy A., Litonjua A.A., Himes B.E., Lange C., Lazarus R., Sylvia J., Klanderman B. (2011). Genomewide association between GLCCI1 and response to glucocorticoid therapy in asthma. N. Engl. J. Med..

[95] Weinshilboum R.M., Sladek S.L. (1980). Mercaptopurine pharmacogenetics: Monogenic inheritance of erythrocyte thiopurine methyltransferase activity. Am. J. Hum. Genet..

[96] Yang S.K., Hong M., Baek J., Choi H., Zhao W., Jung Y., Haritunians T., Ye B.D., Kim K.J., Park S.H. (2014). A common missense variant in NUDT15 confers susceptibility to thiopurine-induced leukopenia. Nat. Genet..

[97] Moriyama T., Nishii R., Perez-Andreu V., Yang W., Klussmann F.A., Zhao X., Lin T.N., Hoshitsuki K., Nersting J., Kihira K. (2016). NUDT15 polymorphisms alter thiopurine metabolism and hematopoietic toxicity. Nat. Genet..

[98] Colombel J.F., Ferrari N., Debuysere H., Marteau P., Gendre J.P., Bonaz B., Soule J.C., Modigliani R., Touze Y., Catala P. (2000). Genotypic analysis of thiopurine S-methyltransferase in patients with Crohn’s disease and severe myelosuppression during azathioprine therapy. Gastroenterology.

[99] Cuffari C., Hunt S., Bayless T. (2001). Utilisation of erythrocyte 6-thioguanine metabolite levels to optimise azathioprine therapy in patients with inflammatory bowel disease. Gut.

[100] Dubinsky M.C., Yang H., Hassard P.V., Seidman E.G., Kam L.Y., Abreu M.T., Targan S.R., Vasiliauskas E.A. (2002). 6-MP metabolite profiles provide a biochemical explanation for 6-MP resistance in patients with inflammatory bowel disease. Gastroenterology.

[101] Schwab M., Schaeffeler E., Marx C., Zanger U., Aulitzky W., Eichelbaum M. (2001). Shortcoming in the diagnosis of TPMT deficiency in a patient with Crohn’s disease using phenotyping only. Gastroenterology.

[102] Asada A., Nishida A., Shioya M., Imaeda H., Inatomi O., Bamba S., Kito K., Sugimoto M., Andoh A. (2016). NUDT15 R139C-related thiopurine leukocytopenia is mediated by 6-thioguanine nucleotide-independent mechanism in Japanese patients with inflammatory bowel disease. J. Gastroenterol..

[103] Zhu X., Wang X.D., Chao K., Zhi M., Zheng H., Ruan H.L., Xin S., Ding N., Hu P.J., Huang M. (2016). NUDT15 polymorphisms are better than thiopurine S-methyltransferase as predictor of risk for thiopurine-induced leukopenia in Chinese patients with Crohn’s disease. Aliment Pharmacol. Ther..

[104] Walker G.J., Harrison J.W., Heap G.A., Voskuil M.D., Andersen V., Anderson C.A., Ananthakrishnan A.N., Barrett J.C., Beaugerie L., Bewshea C.M. (2019). Association of Genetic Variants in NUDT15 With Thiopurine-Induced Myelosuppression in Patients With Inflammatory Bowel Disease. JAMA.

[105] Heap G.A., Weedon M.N., Bewshea C.M., Singh A., Chen M., Satchwell J.B., Vivian J.P., So K., Dubois P.C., Andrews J.M. (2014). HLA-DQA1-HLA-DRB1 variants confer susceptibility to pancreatitis induced by thiopurine immunosuppressants. Nat. Genet..

[106] Wilson A., Jansen L.E., Rose R.V., Gregor J.C., Ponich T., Chande N., Khanna R., Yan B., Jairath V., Khanna N. (2018). HLA-DQA1-HLA-DRB1 polymorphism is a major predictor of azathioprine-induced pancreatitis in patients with inflammatory bowel disease. Aliment Pharmacol. Ther..

[107] Heap G.A., So K., Weedon M., Edney N., Bewshea C., Singh A., Annese V., Beckly J., Buurman D., Chaudhary R. (2016). Clinical Features and HLA Association of 5-Aminosalicylate (5-ASA)-induced Nephrotoxicity in Inflammatory Bowel Disease. J. Crohns Colitis.

[108] Cleynen I., Van Moerkercke W., Billiet T., Vandecandelaere P., Vande Casteele N., Breynaert C., Ballet V., Ferrante M., Noman M., Assche G.V. (2016). Characteristics of Skin Lesions Associated With Anti-Tumor Necrosis Factor Therapy in Patients With Inflammatory Bowel Disease: A Cohort Study. Ann. Intern. Med..

[109] Tillack C., Ehmann L.M., Friedrich M., Laubender R.P., Papay P., Vogelsang H., Stallhofer J., Beigel F., Bedynek A., Wetzke M. (2014). Anti-TNF antibody-induced psoriasiform skin lesions in patients with inflammatory bowel disease are characterised by interferon-γ-expressing Th1 cells and IL-17A/IL-22-expressing Th17 cells and respond to anti-IL-12/IL-23 antibody treatment. Gut.

[110] Johnson J.A., Caudle K.E., Gong L., Whirl-Carrillo M., Stein C.M., Scott S.A., Lee M.T., Gage B.F., Kimmel S.E., Perera M.A. (2017). Clinical Pharmacogenetics Implementation Consortium (CPIC) Guideline for Pharmacogenetics-Guided Warfarin Dosing: 2017 Update. Clin. Pharmacol. Ther..

[111] Davila-Fajardo C.L., Diaz-Villamarin X., Antunez-Rodriguez A., Fernandez-Gomez A.E., Garcia-Navas P., Martinez-Gonzalez L.J., Davila-Fajardo J.A., Barrera J.C. (2019). Pharmacogenetics in the Treatment of Cardiovascular Diseases and Its Current Progress Regarding Implementation in the Clinical Routine. Genes.

[112] Weersma R.K., Xavier R.J., Consortium I.B.D.M.O., Vermeire S., Barrett J.C. (2018). Multiomics Analyses to Deliver the Most Effective Treatment to Every Patient With Inflammatory Bowel Disease. Gastroenterology.

[113] Martin J.C., Boschetti G., Chang C., Ungaro R., Giri M., Chuang L.-S., Nayar S., Greenstein A., Dubinsky M., Walker L. (2018). Single-cell analysis of Crohn’s disease lesions identifies a pathogenic cellular module associated with resistance to anti TNF therapy. bioRxiv.

[114] Kinchen J., Chen H.H., Parikh K., Antanaviciute A., Jagielowicz M., Fawkner-Corbett D., Ashley N., Cubitt L., Mellado-Gomez E., Attar M. (2018). Structural Remodeling of the Human Colonic Mesenchyme in Inflammatory Bowel Disease. Cell.

[115] Uniken Venema W.T., Voskuil M.D., Vila A.V., van der Vries G., Jansen B.H., Jabri B., Faber K.N., Dijkstra G., Xavier R.J., Wijmenga C. (2019). Single-Cell RNA Sequencing of Blood and Ileal T Cells From Patients With Crohn’s Disease Reveals Tissue-Specific Characteristics and Drug Targets. Gastroenterology.

[116] Parikh K., Antanaviciute A., Fawkner-Corbett D., Jagielowicz M., Aulicino A., Lagerholm C., Davis S., Kinchen J., Chen H.H., Alham N.K. (2019). Colonic epithelial cell diversity in health and inflammatory bowel disease. Nature.

